# Pan-cancer classification by regularized multi-task learning

**DOI:** 10.1038/s41598-021-03554-8

**Published:** 2021-12-20

**Authors:** Sk Md Mosaddek Hossain, Lutfunnesa Khatun, Sumanta Ray, Anirban Mukhopadhyay

**Affiliations:** 1 Computer Science and Engineering, Aliah University, Kolkata, 700160 India; 2 Computer Science and Engineering, University of Kalyani, Kalyani, 741235 India

**Keywords:** Computational models, Machine learning, Computational biology and bioinformatics, Data mining

## Abstract

Classifying pan-cancer samples using gene expression patterns is a crucial challenge for the accurate diagnosis and treatment of cancer patients. Machine learning algorithms have been considered proven tools to perform downstream analysis and capture the deviations in gene expression patterns across diversified diseases. In our present work, we have developed PC-RMTL, a pan-cancer classification model using regularized multi-task learning (RMTL) for classifying 21 cancer types and adjacent normal samples using RNASeq data obtained from TCGA. PC-RMTL is observed to outperform when compared with five state-of-the-art classification algorithms, viz. SVM with the linear kernel (SVM-Lin), SVM with radial basis function kernel (SVM-RBF), random forest (RF), k-nearest neighbours (kNN), and decision trees (DT). The PC-RMTL achieves 96.07% accuracy and 95.80% MCC score for a completely unknown independent test set. The only method that appears as the real competitor is SVM-Lin, which nearly equalizes the accuracy in prediction of PC-RMTL but only when complete feature sets are provided for training; otherwise, PC-RMTL outperformed all other classification models. To the best of our knowledge, this is a significant improvement over all the existing works in pan-cancer classification as they have failed to classify many cancer types from one another reliably. We have also compared gene expression patterns of the top discriminating genes across the cancers and performed their functional enrichment analysis that uncovers several interesting facts in distinguishing pan-cancer samples.

## Introduction

Cancer is a generic term that indicates a broad array of disorders that may impact any region of our human body. It is the second major cause of death all over the globe. In cancer, malignant tumors or abnormal cells grow abundantly with the potential to infect other cells of the body through the bloodstream. Throughout the last few decades, there has been continuous development in cancer research. Researchers applied numerous methodologies for screening out preliminary cancer stages to determine cancer types even before they become symptomatic. The evolution of modern technologies in bioinformatics brings forth a massive surge in the collection and availability of cancer and other diversified diseases’ data to scientists. Applications of machine learning algorithms possess the immense capability to analyze such a massive amount of data^1^ and it has been extensively used in sub-classification of diseases, gene identification problems, studying diseases’ progression characteristics, etc^2–8^. The advancement of recent next-generation RNA Sequencing technology (RNASeq) lay the foundation for The Cancer Genome Atlas (TCGA), a multi-platform cancer data repository with more than 11,000 patients and 33 cancer types. This massive amount of data instigate the uncovering of tumorigenic features by examining the difference between tumorous and non-tumorous data. It also makes it possible to reveal key features that differentiate among the cancer types and comprehend poorly examined cancers.

Many researchers in the recent past have utilized the classification of cancer types using transcriptomic profiles to overcome the limitations of diagnostic capabilities of conventional clinical and morphological approaches^9,10^. Danaee et al.^11^ applied Stacked Denoising Autoencoder (SDAE) to discern genes most relevant for diagnosing breast cancer. SDAE was used here for feature selection, identifying deeply connected genes (DCG), i.e., genes having significant interactions among themselves from the RNASeq gene expressions dataset in breast cancer. They utilized single-layer ANN, SVM with the linear kernel (SVM-Lin), SVM with radial basis function kernel (SVM-RBF) to distinguish tumor samples from normal samples. In this binary classification problem, the best classification was achieved using SVM-RBF, which yields 94.78% accuracy. In^12^, the genetic algorithm was used to detect several subsets of 20 genes for pan-cancer classification of 32 cancer types using RNASeq data by the k-nearest neighbors (kNN) classifier. However, their technique was computationally expensive as they ran the GA/kNN thousand iterations to discover the gene sets, and classification accuracy was inconsistent across the cancers. Kim et al.^13^ performed classification of 21 cancer types using SVM-Lin, SVM-RBF, kNN, random forest (RF), and neural network (NN) classifiers with 300 most significant differentially expressed (DE) genes detected by applying ANOVA on RNASeq gene expressions data collected from TCGA. They attained the best results with NN that yields accuracy: 0.9 and Matthews Correlation Coefficient (MCC): 0.89. Nevertheless, with the NN classification model, several cancer types were incorrectly classified as one another, especially in classifying squamous cells carcinomas (CESC, LUSC, HNSC), subtypes of lung cancer (LUAD, LUSC), cancers in kidney tissues (KICH, KIRC, and KIRP), CHOL, COAD, READ, ESCA, PAAD, STAD, and BLCA.

Multi-task learning (MTL) mines association among a set of related tasks to improve classification performance^14^. Although less studied than other machine learning models, MTL is extremely powerful in several data-intensive applications where training samples of the learning tasks have high degrees of similarity. MTL has been utilized in diversified fields including image processing and computer vision^15^, web search ranking^16^, deep learning-based natural language processing^17^, text classification^18^ and biomedical researches^19–21^.

In the present article, we leveraged the landmark advantages of regularized multi-task learning (RMTL)^22^ and developed PC-RMTL, a classification model for the pan-cancer classification task. Here, we have initially applied DESeq2 to evaluate the differential gene expression to identify highly significant differentially expressed (DE) genes and perform dimensionality reduction for each cancer. The union-set of all those DE genes has been used to perform pan-cancer classifications among 21 cancer types and adjacent normal samples using SVM-Lin, SVM-RBF, kNN, RF, decision tree (DT), and PC-RMTL. All the classifiers’ performance has been compared through precision, recall, f1-score, Matthews correlation coefficient (MCC), ROC curve, precision–recall curve, and logistic loss. Despite the highly imbalanced nature of the pan-cancer dataset (due to significant differences in the number of samples for each cancer type), PC-RMTL outperforms all the other five classifiers achieving 96.07% accuracy and 95.80% MCC score. PC-RMTL performs exceptionally well in distinguishing all of the pan-cancer samples except the READ. SVM-Lin and SVM-RBF have shown highly comparable prediction results with PC-RMTL. We have compared all the competing methods’ classification performance with a small group of features (genes) that have been detected using coefficients (weights) of the trained linear SVM (SVM-Lin) and a widely used independent features selection algorithm called ‘minimum redundancy maximal relevance' (MRMR)^23^. PC-RMTL again outperformed all the competing classifiers for all those small sets of selected features. We have further analyzed the discriminating capability of the selected features (genes) using functional enrichment analysis. It has been discovered that almost all of the top discriminating genes are highly associated with cancer-related gene ontology (GO) terms and pathways.

*Summary of contributions* In this work, we have made the following novel contributions:

(1): We have explored the first multi-task learning model to classify 21 pan-cancers and adjacent normal samples of TCGA data. We have employed the regularized version of multi-task learning (MTL) that has a significant advantage over single-task learning, enabling us to learn several tasks (here, several cancer data) simultaneously. RNASeq expression data of multiple cancers has been utilized in a mutual context via a notion of relatedness of all the tasks. Here, the objective of each task is to learn the RNASeq gene expression patterns of the tumor samples of a particular cancer type.
(2): Our approach is the first to explicitly address how to learn the feature representation of multiple cancer types’ samples simultaneously. We have raised an objective function (see Eq. 2) to minimize the logistic loss of the classification process.
(3): Our framework (with tuned parameters) can classify unseen RNASeq expression data with utmost accuracy. Classifying hitherto unclassified cancer samples is crucial for early diagnosis of the disease. We present our framework to be effective in this case. We demonstrate that the model can classify completely unseen test samples with high accuracy.
(4): It is challenging to obtain essential regulatory genes that can be utilized to discriminate different cancer samples. Here, we demonstrate 75 genes having useful discriminating features.

**Figure 1:**
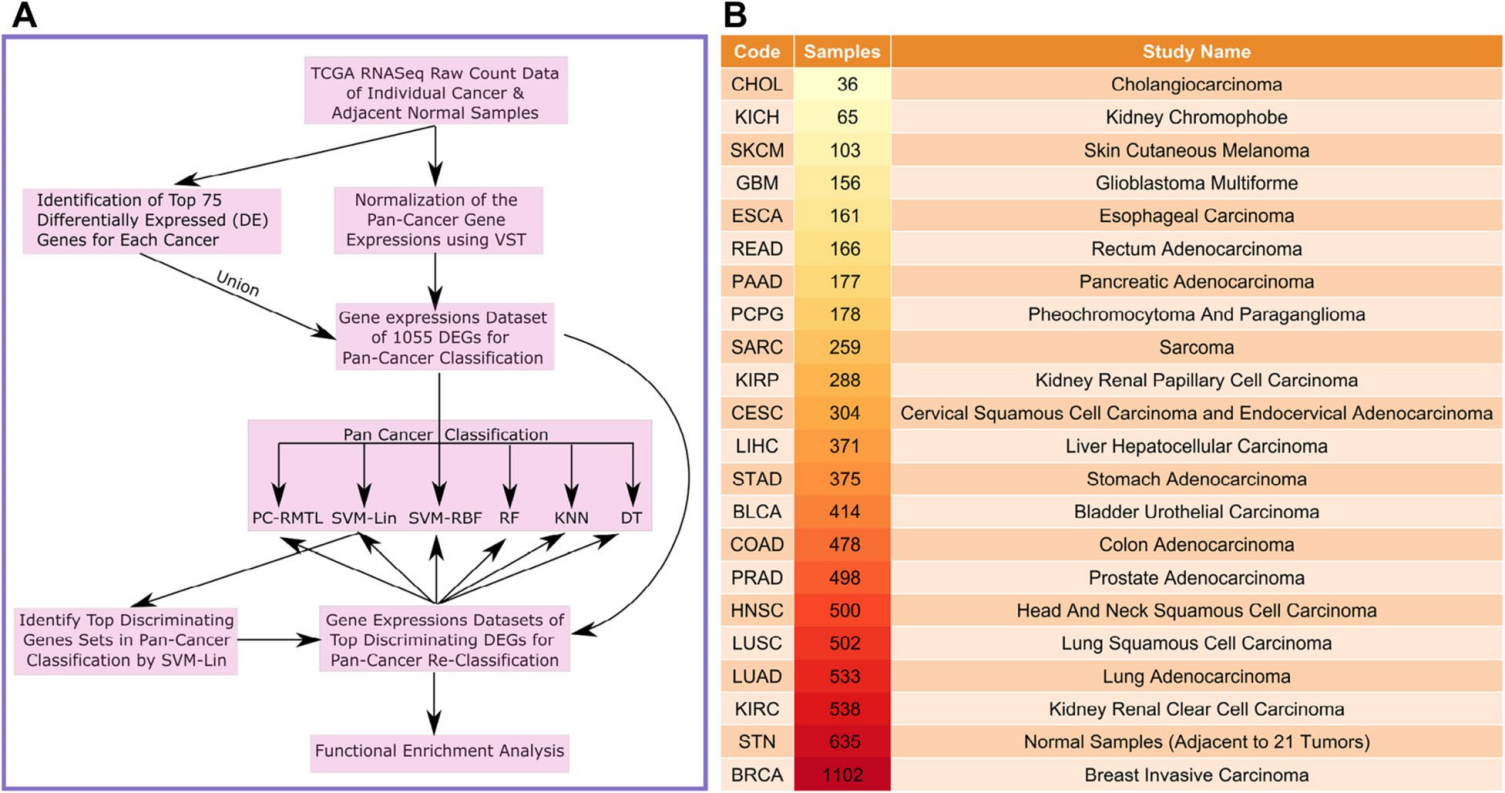
The figure shows (**A**) the overall framework for the classification model and (**B**) TCGA cancer types and the number of RNASeq samples used in this study.

## Materials and methods

### Preparing the data

In our experiments, we have used cancer samples of 21 different types along with their adjacent normal tissue samples. We have obtained RNASeq transcript abundance counts of 56493 Ensembl genes for 21 cancer types from TCGA data portal. The R/Bioconductor package TCGAbiolinks^24^ has been utilized to retrieve the raw count data from the GDC cancer data portal. Figure 1B shows a detailed description of the cancer types used here. Initially, we have identified the top 75 differentially expressed (DE) genes (adjusted *p* value $\le 0.05$ and fold-change $\ge 2$) using DESeq2 R package^25^, for each cancer type by comparing the tumor and normal samples. Next, we have obtained 1055 highly significant genes (HUGO gene symbols) from the union set of those top 75 DE genes selected in the previous step. Thus the identified genes are differentially expressed in at least one of the cancer types. Mapping from Ensembl gene ids to HUGO gene symbols was performed via the biomaRt^26^ R/Bioconductor package. Variance stabilizing transformation (VST)^27^ has been used to obtain normalized gene-expression values from the raw transcript abundance counts. Here, we have considered only primary tumor and adjacent normal samples of all the 21 cancer types for the pan-cancer classification task. Thus, among the 7839 collected samples with 22 sample classes, 21 classes correspond to 21 different primary tumor samples and one normal sample class that includes all the normal samples from each cancer type (Fig. 1B). In the following sections, we first describe our analysis pipeline and the fundamental ideas supporting it.

### Workflow

In Fig. 1A, we have described the workflow of our analysis pipeline. All of the essential steps are discussed in this subsection.

(1) *Collecting TCGA RNASeq raw counts of* 21 *cancer types* Initially, we have obtained RNASeq transcript abundance counts of 56493 Ensembl gene ids for 21 cancer types from TCGA, which was utilized earlier in pan-cancer classification by Kim et al.^13^. We have considered the primary tumor and the adjacent normal tissue samples of those 21 cancer types.

(2) *Computing the common set of DE genes* We have identified the differentially expressed (DE) genes for each cancer type using the R/Bioconductor package DESeq2^25^. DESeq2 finds out the DE genes by comparing the transcript abundance between the primary tumor and the normal samples. We have identified 1055 highly significant genes (HUGO gene symbols) by taking a union among the individual sets of the top 75 DE genes detected for each cancer type.

(3) *Classifying samples using PC-RMTL and other state-of-the-art classifiers* PC-RMTL and other state-of-the-art classifiers are trained with the VST normalized gene expressions of the identified DE genes to classify 21 cancer types and adjacent normal samples. PC-RMTL utilizes a regularized multi-task learning (RMTL) model with $L_{2,1}$ regularization technique for learning the 22 task simultaneously. Here, we have partitioned the whole classification dataset comprising of 7839 samples with an 8:2 train-test ratio using the stratified random sampling technique. To discover the best hyperparameters for each classification model, we have performed 10-fold cross-validation repeated 10 times with distinct values of the hyperparameters using only the training samples. Later, the trained models with best hyperparameters have been evaluated with the completely independent test samples.

(4) *Identifying the top discriminating DE genes* We have identified the top key discriminating features (genes) using the coefficients (weights) of the trained linear SVM (SVM-Lin). Once an SVM-Lin model is trained with the training samples, the coefficients (weights) of the fitted model can be obtained that represent the vector coordinates orthogonal to the hyperplane, and their direction determines the predicted class. By comparing the absolute size of the coefficients, it is possible to discover the importance of all the features of a classification model. Moreover, to make a fair comparison, we have also utilized a widely used independent features (genes) selection algorithm called ‘minimum redundancy maximal relevance' (MRMR)^23^, for comparing the performance of PC-RMTL with other competing methods in smaller datasets (data with a small number of selected features: genes). We have gradually identified the top 75, 100, 200, 300, 400, 500, 600, 700, 800, 900, 1000 discriminating features (genes) accountable for pan-cancer classification.

(5) *Functional enrichment analysis of the top discriminating DE genes* After identifying the essential discriminating DE genes in pan-cancer classification using SVM-Lin, we have compared gene expressions of those top discriminating genes and performed their functional enrichment analysis.

(6) *Classification with small features (genes)* We have also performed the classification task using several small sets of top discriminating DE genes discovered from the SVM-Lin classifier and the MRMR^23^ feature selection algorithm. It has been observed that the PC-RMTL outperforms all other classification models with these small sets of features.

### Multi-task learning

The statistical learning approach for multi-task learning (MTL) was first introduced in the article^28^ to choose an optimal hypothesis space from a family of hypothesis spaces. In^29^, the notion of the “extended VC dimension” was introduced to compute the bounds on the average error of *T* tasks. The same framework was utilized in^28^ to model the amount of information needed to learn a task using Bayesian and information theory arguments. In^30^, the extended VC dimension was utilized to derive strict tighter bounds for each task, assuming that the learning tasks are related in a particular way.

In general, MTL deals with multiple learning tasks, including general learning such as supervised tasks (e.g., classification problems, regression problems), unsupervised tasks (e.g., clustering problems), reinforcement learning tasks, semi-supervised tasks, etc. Among all these, it is assumed that all tasks or a subset of the tasks are related. The main advantage of MTL is that it can jointly learn tasks that can leads to much performance improvement compared with individual learning tasks. Hence, the main aim of applying MTL is to improve the generalization performance of multiple related tasks.

The idea behind the methodologies of multi-task learning is that given *T* learning tasks, assuming that data for all the tasks come from the same space of $X \times Y$, the conditional distribution of the response variable (*Y*: $Y_t|X_t$, for all *t*) are related, where *X* is the explanatory variable of all *T* tasks. In particular, given *t* learning tasks: ${\tau }_{i=1}^{t}$, we have *n* data points: $(x_{1t}, y_{1t}), (x_{2t}, y_{2t}), \ldots, (x_{nt}, y_{nt})$ for each $\tau _i$, where the data point coming from a distribution $P_t$ on $X \times Y$, $P_t$ is different for each task but MTL assumes that $P_t$ of all tasks are related. Here, the aim is to learn *t* functions $f_1,f_2, \ldots, f_t$, each for a learning task, such that $f_t(x_{it})=y_{it}$. When $t=1$, the problem reduces to single-task learning. Several setups may be possible for the MTL problem. One of the simpler versions is when the input data $x_{it}$ is the same for all tasks. In that case although the output value $y_{it}$ differs from each other, $x_{it}$ remains same for all *t*. The other scenario may be the case of having the same output $y_{it}$ for different inputs $x_{it}$, which corresponds to the problem of integrating information from heterogeneous databases^31^.

### Regularized multi-task learning for PAN cancer classification

In^22^, a regularization-based approach is proposed to solve the MTL problem, where the regularization functions are minimized analogously to SVM used in single-task learning. All the MTL algorithms more or less try to minimize the following objective function:

1 $$\begin{aligned} \min _{W,C} \sum _{i=1}^t {\mathscr{L}}(W_i,C_i|X_i,Y_i) + \lambda _1 \omega (W) + \lambda _2||W||^2_F, \end{aligned}$$

where $L({\circ })$ represents the loss function, $\omega$ represents the cross-task regularization, $\lambda _1$ and $\lambda _2$ are positive regularization parameters. $\lambda _1$ signifies the strength of relatedness of all tasks and is estimated through a cross-validation procedure, whereas $\lambda _2$ is to introduce the penalty of the quadratic form of *W*. Here, *F* is the Frobenius norm. $\lambda _2$ promotes the selection of correlated predictors, stabilizes the results, and improves the generalization performance. The term $\omega (W)$ transfer knowledge across the tasks with a specific regularization technique that jointly modulates multi-task models $({W_1, W_2, \ldots , W_t})$ in accordance with precise prior structure of *W* and the vector $C = [c_t] ; c_t \in {\mathbb {R}}$ contains constants associated with all tasks.

In our present work, we have developed a pan-cancer classification model (PC-RMTL), incorporating the $L_{2,1}$ regularization for joint feature selection with the objective function^32–34^:

2 $$\begin{aligned} \min _{W,C} \sum _{i=1}^t {\mathscr{L}}(W_i,C_i|X_i,Y_i) + \lambda _1 \left\| W_{2,1} \right\| + \lambda _2 \left\| W \right\| ^2_F \end{aligned}$$

Here, $L(\circ )$ in Eq. (2) is the logistic loss function for classification defined as:

3 $$\begin{aligned} {\mathscr{L}}(W_i, C_i) = \frac{1}{n} \sum _{k = 1}^{n} \log (1 + e^{-Y_{i,k}(X_{i,k}W_i^T + C_i)}), \end{aligned}$$

where *i* indices tasks and *k* indices samples in each task. Therefore $Y_{i,k}$ and $X_{i,k}$ refer to the outcome and predictors of subject *k* in task *i*, while *n* refer to the number of subjects in task *i*. Once the objective function defined in Eq. (2) is optimized, the coefficient matrices: *W*, *C* of all tasks are estimated, and the learning algorithm becomes capable of predicting the class labels of unknown observations.

In our PC-RMTL model, the goal of each task is to learn the expression matrices ($M_1,M_2, \ldots, M_t$) of a particular type of samples (*T*). There are 22 types of samples (classes) in our work, 21 different cancer types, and one type of sample comprising all the normal tissue samples adjacent to each type of cancer. In this work, we have formulated the pan-cancer RNASeq gene expression dataset for classification in such a way that share the identical predictor matrices: $\{X_i, i \in {1, 2, \ldots, t}\}$ for all the learning tasks, but each of the response vectors $\{Y = {Y_i}; i \in {1, 2, \ldots, t}\}$ is different. In particular, we have combined the RNASeq gene-expression data from all the 22 types of samples (classes) to prepare the predictor matrix $X_i$. This predictor matrix is the same for all the tasks. The response vector for a specific task corresponding to a particular class contains $+1$ for samples belonging to that class, $-1$, otherwise. The response vectors for all the tasks are different. Therefore, we have represented each task as:

4 $$\begin{aligned} S_i = \{(X_i, Y_i), X_i \in R^{n \times p}, Y_i \in \{1,-1\}^n\} , \end{aligned}$$

where, $X_i = [M_1; M_2; \ldots ; M_i; \ldots ; M_t]; M_i \in {\mathbb {R}}^{n_i \times p} \; \text {and} \; X_i \in {\mathbb {R}}^{n \times p}$, $n_i$ represents number of samples in each class *i*, $n = \sum _{i = 1}^{t} n_i$ represents the number of samples across all the classes and *p* represents the number of selected differentially expressed (DE) genes from RNASeq expression data.

### Description of the state-of-the-art methods

To verify the effectiveness of the PC-RMTL for pan-cancer classification, we have utilized five state-of-the-art classification algorithms, viz. SVM-Lin, SVM-RBF, kNN, RF and DT. All classification algorithms have been trained using the training samples constructed by partitioning the whole classification dataset comprising of 7839 samples with an 8 : 2 train-test ratio using the stratified random sampling technique.

#### Parameters and settings for comparisons

We have comprehensively examined the performance of all the competitive classifiers using several combinations of hyperparameters and selected those that give the best performance. In SVM-Lin: the regularization parameter $C = 0.01$ and in SVM-RBF: $C = 10$, $gamma = 0.0001$ provides the best performance. Similarly, it has been observed that in kNN classifier $k = 6$, while in decision tree (DT) and random forest (RF), $min\_samples\_leaf$: 11 and 2 and $min\_samples\_split$: 22 and 18, respectively provide best performance. RF has been built with 100 trees to produce the best results. In PC-RMTL, $\lambda _1 = 0.0001$ and $\lambda _2 = 0.00001$ provides best classification results. To discover the best hyperparameters for each classification model, we have performed 10-fold cross-validation repeated 10 times with different values of the hyperparameters using only the training samples. Later with the determined best parameters of the classification models, each classifier trained afresh using the whole-lot of training samples and performance of the models was evaluated using the completely independent test samples.

### Performance evaluation of the classification models

To classify unknown samples, PC-RMTL yields the probability of a sample to be present in a positive class $(P(Y == 1))$. The unknown sample is assigned the class label with the highest probability (> 0.5). To evaluate the performance of the different classification models, we have used the following metrics: accuracy (ACC), precision, recall, and f1-score. To make a fair comparison in highly imbalanced data (which is our case), we have computed the Matthews correlation coefficient (MCC), which imparts a high score only if the prediction provides good results in all four confusion matrix categories (true positives (TP), false positives (FP), true negatives (TN) and false negatives (FN)). In multi-class classification, the MCC can be computed from the confusion matrix (*C*) for *M* classes and is defined as:

5 $$\begin{aligned} MCC = \frac{n_c \times n_s - \sum _{j}^{M}{p_j \times t_j}}{\sqrt{(n_s^2 - \sum _{j}^{M}{p_j^2}) \times (n_s^2 - \sum _{j}^{M}{t_j^2})}}, \end{aligned}$$

where $t_j = \sum \nolimits _{i}^{M}C_{ij}$: number of times class *k* truly occurs, $p_j = \sum \nolimits _{i}^{M}C_{ji}$: number of times class *k* is predicted, $n_c = \sum \nolimits _{j}^{M}C_{jj}$: number of samples correctly predicted, $n_s = \sum \nolimits _{i}^{M}\sum _{k}^{M} C_{ik}$}: number of samples.

In the case of multi-class classification, the minimum value of MCC ranges between $-1$ and 0 according to the number and distribution of true class labels while the maximum value is $+1$.

We have also compared logistic regression loss (log loss) or cross-entropy loss for all the classification models to evaluate the probability outputs of the classifiers instead of their discrete predictions. A perfect model with all the samples correctly predicted has a log loss of 0. Let the actual labels for a set of samples be encoded as a 1-of-*M* binary indicator matrix *M*, i.e., $y_{j,m} = 1$, if sample *j* has label *m* taken from a set of *M* labels; *P* denotes the probability estimator matrix, with $p_{j,m} = Pr(y_{j,m} = 1)$. Then the log loss for the entire set of $n_s$ test samples is defined as:

6 $$\begin{aligned} {\mathscr{L}}_{\log _e(Y,P)}= & {} - \log _e Pr(Y, P) \end{aligned}$$

7 $$\begin{aligned}= & {} - \frac{1}{n_s} \sum _{j = 1}^{n_s} \sum _{m = 1}^{M} y_{j, m} \; log_e \; p_{j, m} . \end{aligned}$$

### Functional enrichment analysis of top discriminating genes

We have identified the key discriminating DE genes in the pan-cancer classification task using the coefficients (weights) of the trained SVM-Lin model. These top discriminating genes are then utilized to classify the samples again. We have also compared gene expressions of the top discriminating genes and performed their functional enrichment analysis. The top 10 most significant (according to lowest “*p* value”) gene ontology terms (biological processes), KEGG pathways, and disease-genes associations of those top discriminating genes have been identified through the Enrichr^35^ and the DisGeNET^36^.

## Results and discussion

We have used gene expressions data of the DE genes as an input to the PC-RMTL and other competing methods for classifications. We also demonstrate that PC-RMTL provides better prediction accuracy than the other competing methods with the DE genes and smaller sets of features (genes) identified through the coefficients (weights) of the trained SVM-Lin and the MRMR feature selection algorithm. It provides sound evidence that PC-RMTL can be utilized in the classification task when the expression of a small number of genes is available. Finally, our study describes a comprehensive comparative analysis of the six classification methods to classify cancer samples. The following sections describe the results of the performed experiments.

### Outcomes of pan-cancer classification

**Figure 2:**
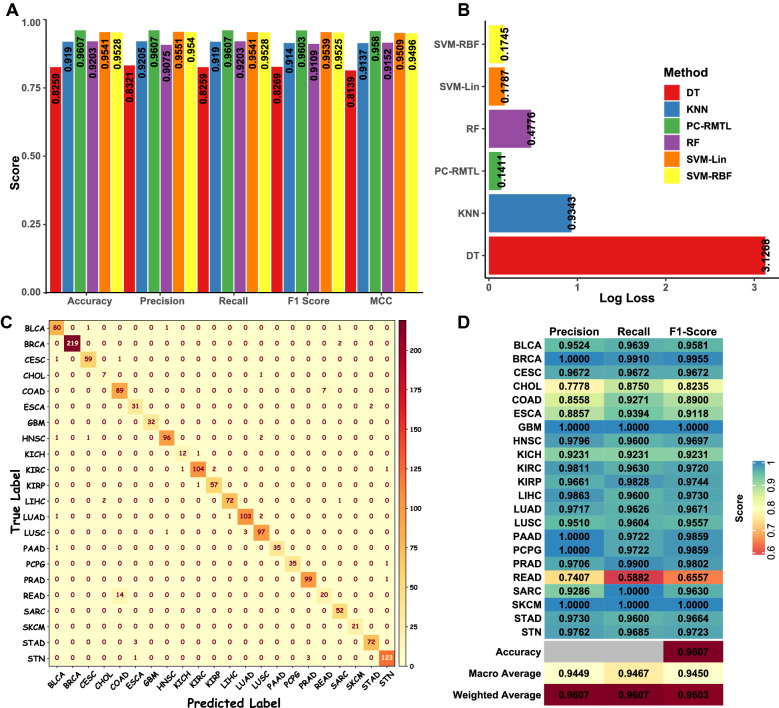
The figure shows the (**A**) classification statistics for all the classifier models using accuracy, precision, recall, f1-score, and MCC metrics, (**B**) logistic loss for each classification model, (**C**) the confusion matrix shows the accuracy of the PC-RMTL model for pan-cancer classification and (**D**) precision, recall, and f1-score of the PC-RMTL classifier for each class.

We have observed that PC-RMTL outperforms all the state-of-the-art classifiers. It can be seen from the Fig. 2A that the highest accuracy (96.07%), precision (96.07%), recall (96.07%), f1-score (96.03%) and MCC (95.80%) is achieved using PC-RMTL. Only SVM-Lin and SVM-RBF have a tight competition with PC-RMTL, while the performance of DT is not satisfactory compared to other classifiers. Logistic loss exhibited by each of the classifiers is depicted in Fig. 2B, which also proves that PC-RMTL outperforms all other classifiers with a minimum logistic loss.

The confusion matrix in Fig. 2C shows the accuracy of the PC-RMTL classifier for the pan-cancer classification task. It can be observed from the figure that except for the READ cancer, the performance of the PC-RMTL is exceptionally well for distinguishing the pan-cancer samples. Some of the READ samples are misclassified as COAD samples for all of the classifiers, including PC-RMTL. The performance of the RF classifier is the worst in this context as it misclassified all the READ samples. We have also observed that the PC-RMTL has correctly classified 97% of the adjacent normal samples from the pan-cancer samples. On the other hand, 12% of the CHOL samples have been wrongly classified as LUSC samples by the PC-RMTL. This problem might be due to the small number of CHOL tumor samples available in TCGA (only 36 samples) that have been utilized to perform this study.

**Figure 3:**
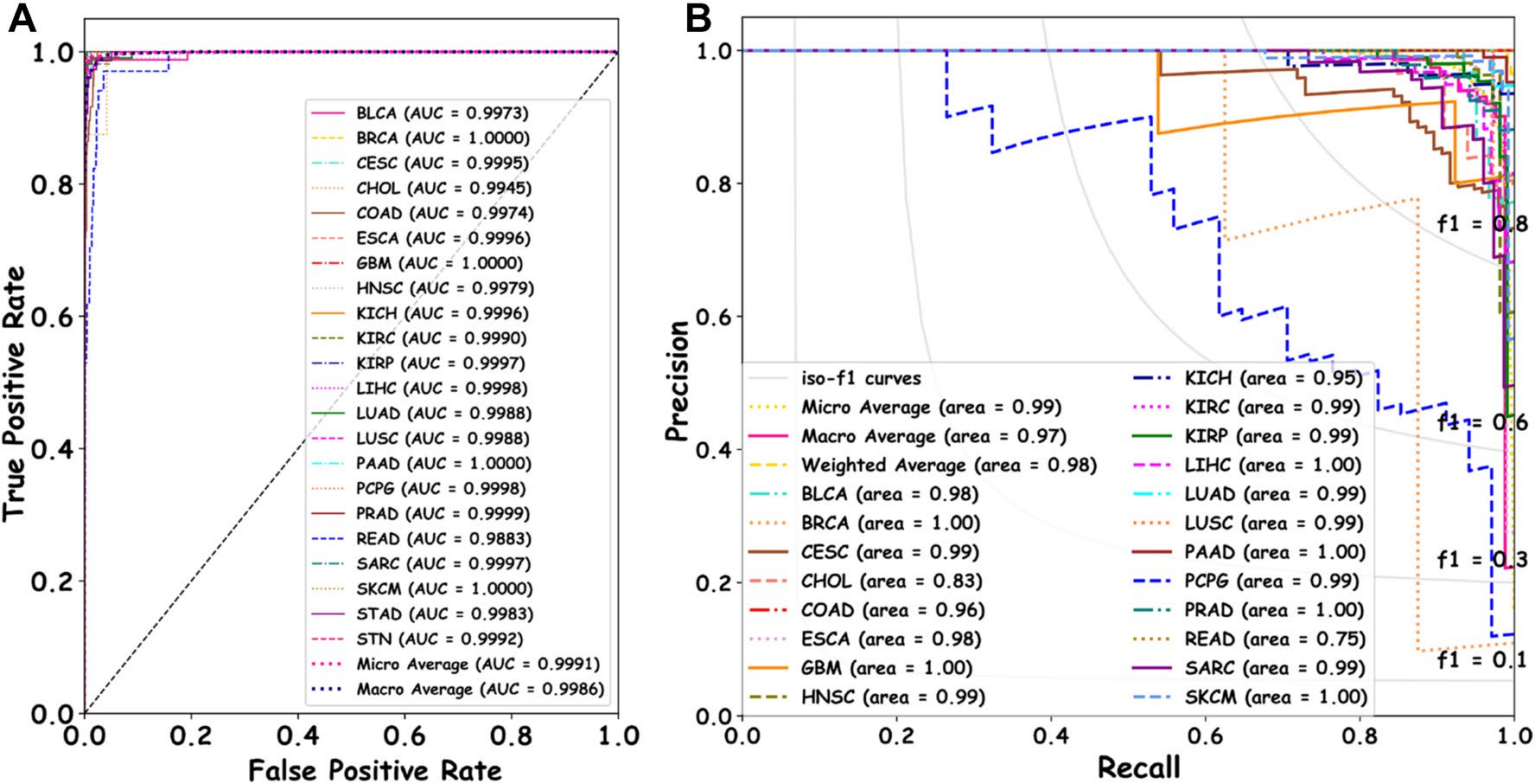
The figure shows the (**A**) ROC curve and (**B**) Precision–Recall curve showing the performance of the PC-RMTL classifier.

Figure 2D shows the performance of the PC-RMTL classifier in predicting the sample labels for individual classes. From this figure, it is clearly visible that except for the READ and CHOL, the PC-RMTL classifier has correctly classified samples in each type of cancer. The supplementary table S1 shows the precision, recall, f1-score of the other competing classifiers. The performance of the PC-RMTL classification model at different classification thresholds is displayed in the Receiver Operating Characteristic (ROC) curve in Fig. 3A. The figure shows the True Positive Rate (TPR) against False Positive Rate (FPR) at different thresholds and the Area Under the Curve (AUC) underneath the entire ROC curve. It also shows that the PC-RMTL performs exceptionally well in distinguishing each of the cancer types except the READ, for which the PC-RMTL exhibit lower AUC (0.9883). Figure 3B shows the precision-recall (PR) curve of the PC-RMTL classifier that summarizes the trade-off between the precision and the recall at different probability thresholds. The PR curve is an effective diagnostic tool for classification models with an imbalanced number of samples in different classes. This figure also confirms that the overall performance of PC-RMTL is excellent in classification tasks.

### Analyzing the top discriminating genes in classification

In our present work, we have observed that although the PC-RMTL outperforms all the state-of-the-art classifiers, SVM-Lin and SVM-RBF have also been able to classify the pan-cancer samples well. This fact tempted us to identify the key discriminating features (genes) in SVM-Lin for pan-cancer samples classification. Once an SVM-Lin model is trained with the training samples, the coefficients (weights) of the fitted model can be obtained that represent the vector coordinates orthogonal to the hyperplane, and their direction determines the predicted class. By comparing the absolute size of the coefficients, it is possible to discover the importance of all the features of a classification model.

**Figure 4:**
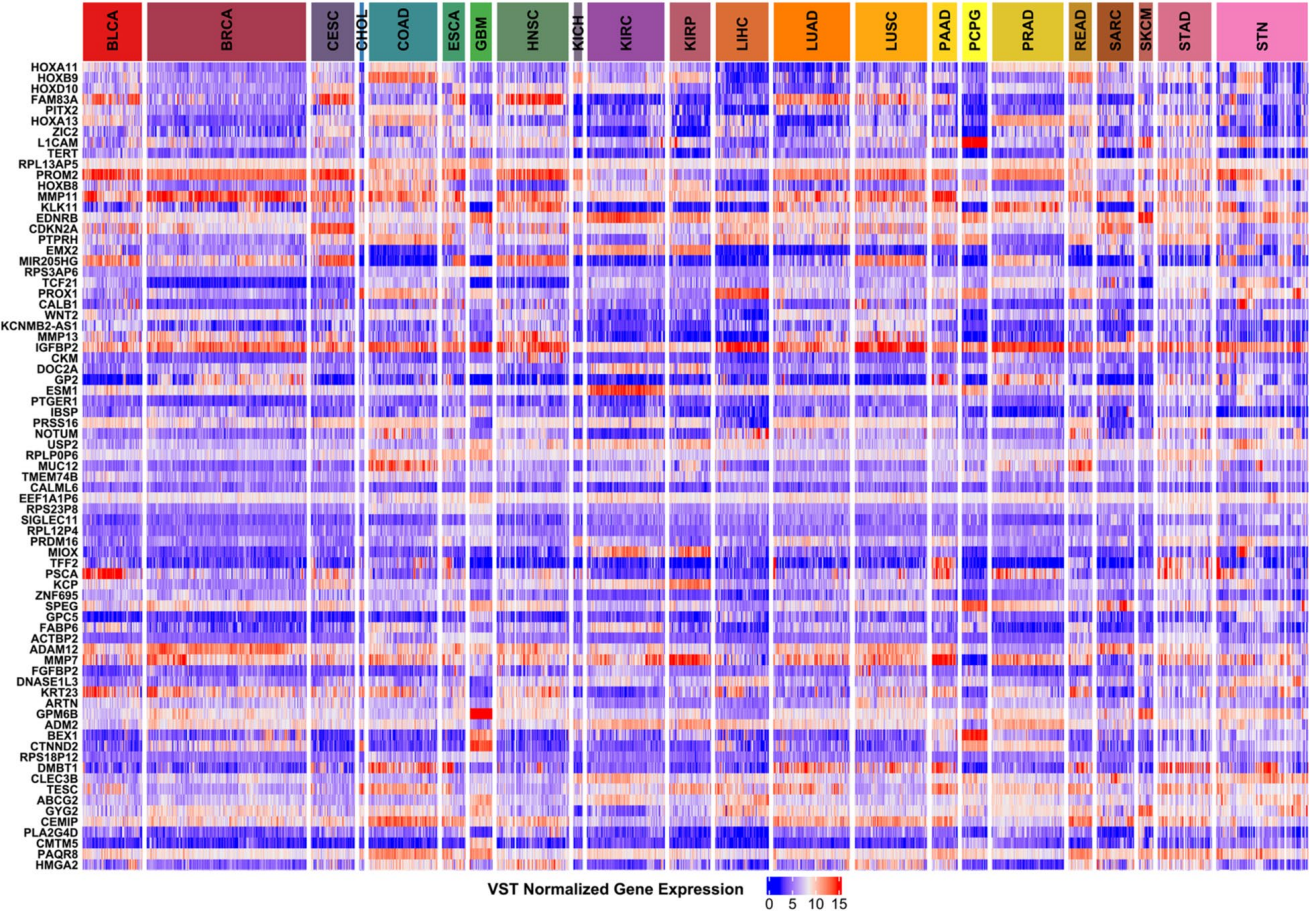
The figure shows the heatmap of gene expressions for the top 75 discriminating genes across the pan-cancers. These top discriminating genes were selected from the SVM-Lin classifier.

We have gradually identified the top 75, 100, 200, 300, 400, 500, 600, 700, 800, 900, 1000 features (genes) accountable for the pan-cancer classification using SVM-Lin. The list of the top 75 genes and their expression pattern across all the samples types are shown in Fig. 4. The figure clearly shows that some genes are highly expressed in one type of cancer samples but witnessed low-expression patterns in others. For example, the genes ‘BEX1’, ‘MUC12’ are highly expressed in GBM-PCPG, and COAD-READ, respectively, but not in other cancers. A thorough analysis of the distinctive capabilities of all the 1055 genes will surely shed light on cancer pathogenesis. Among the top 75 discriminating genes, numerous key regulatory genes in cancers have been detected, e.g. ‘CDKN2A’, ‘TERT’, ‘HMGA2’, ‘ARTN’, ‘MMP7’, ‘ADAM12’, ‘TCF21’, ‘PITX2’, ‘DMBT1’, etc. In most cancers, the genetic abnormalities of ‘CDKN2A’ are reported similar to the ‘p53’ tumor suppressor gene, which is treated as the most commonly mutated gene in human cancer^37^. Similarly, ‘TERT’ gene can be treated as a useful marker in the diagnosis and prognosis of various cancers^38^. ‘TERT’ expression/telomerase activity can be detectable in up to 90% of primary cancers^39^. Considerable evidence has been reported that ‘TERT’ contributes to cancer development and progression via multiple activities^39^.

**Figure 5:**
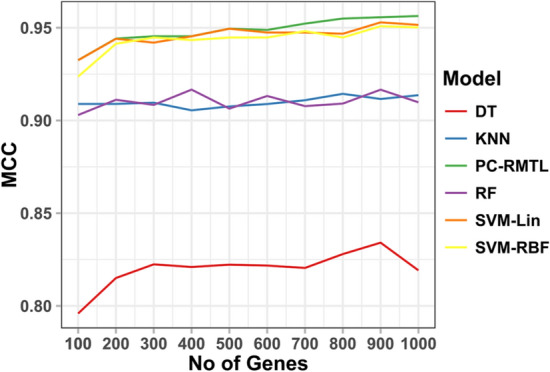
The figure shows the MCC scores vs the number of top features (genes) for each classifier. These top discriminating genes were selected from the SVM-Lin classifier.

Furthermore, we have also performed pan-cancer classification using gene expressions of all those sets of the top discriminating genes discovered from SVM-Lin and repeatedly observed the performance of all the classification models. We have seen that with only 75 and 100 top discriminating genes, respectively, 91.84% and 93.69% pan-cancer classification accuracy have been obtained through the PC-RMTL. Figure 5 shows the performance of all the classification models using those top discriminating genes through MCC scores. It is evident from the figure that although the top discriminating genes have been identified through SVM-Lin, yet PC-RMTL outperforms all the classification models, including SVM-Lin. Moreover, to make a fair comparison, we have also utilized a widely used independent features (genes) selection algorithm called ‘minimum redundancy maximal relevance’ (MRMR)^23^ for comparing the performance of PC-RMTL with other competing methods in smaller datasets (data with a small number of selected features: genes). The supplementary table S2 shows that our PC-RMTL outperformed all other classifiers consistently with top discriminating 100, 200, ..., 1000 features.

**Figure 6:**
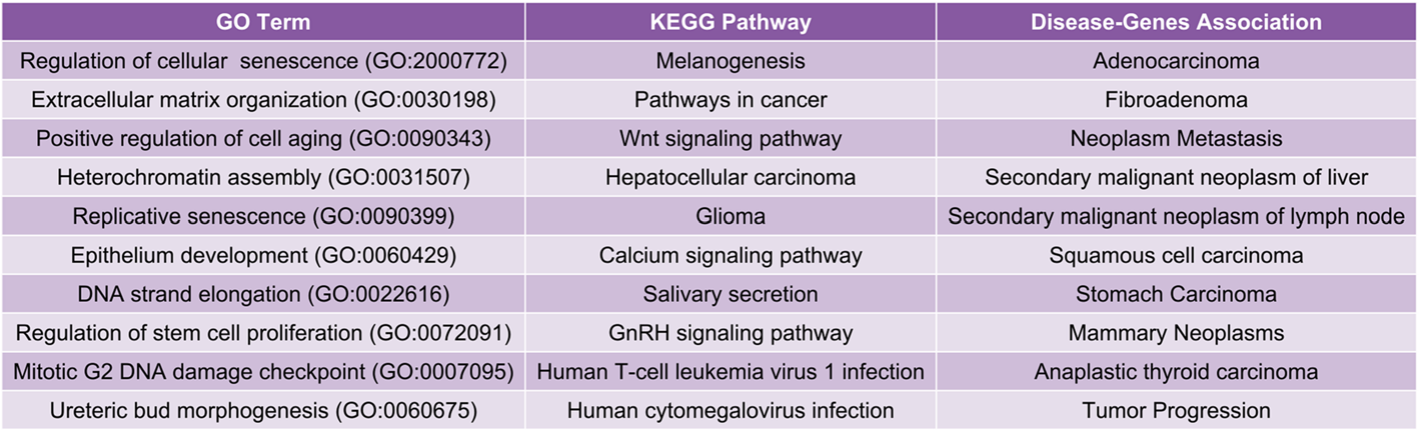
The figure shows the gene ontology terms (biological processes), KEGG pathways, and disease-genes associations of the top 75 discriminating genes selected from the SVM-Lin classifier.

### Functional enrichment analysis of the top discriminating genes

We have also examined the functional roles of the top 75 discriminating genes selected from the SVM-Lin classifier using gene ontology (GO) terms, pathways, and disease-genes associations [Fig. 6, supplementary table S3]. Figure 6 shows that most of the GO terms are related to cell cycle, which is highly associated with tumorigenesis as essentially cancer is induced by uncontrolled cells growth that deregulates cell proliferation, and division^40^. We have also observed that regulation of cellular senescence, stem cell proliferation, extracellular matrix organization, replicative senescence, and DNA damage checkpoint are significant GO terms associated with these genes. Senescence, an essential activity in a cell to protect against malignant transformation, also promotes cancer growth by generating a pro-tumorigenic microenvironment through a senescence-associated secretory phenotype (SASP) by accelerating extracellular matrix remodeling and inflammation^41^. The extracellular matrix is a leading structural component for the tumor microenvironment. Cancer development is closely associated with self-renewal and multipotent differentiation of stem cells. Cancer stem cells initiate cancers and have self-renewal and proliferation, drug-resistant capability, and thus are the driving force of carcinogenesis^42^. DNA damage checkpoints have a critical role in damage repair and act as crucial control points in the cell cycle. Daughter cells lose genomic integrity and accumulate genetic damage due to the loss of checkpoint functions, which are considered the primary reasons for DNA aberrations in cancer. It has also been observed that the top discriminating genes are associated with several cancer-related pathways along with human cytomegalovirus infection, Wnt, calcium, and GnRH signaling, which are key regulators of pathways critical in cancer progression. Human cytomegalovirus infection accelerates damage in the chromosome, thereby promotes genetic instability, which is a major driver in cancer progression^43^. Wnt signaling is a conserved regulatory pathway that controls several normal cellular and developmental processes from an evolutionary perspective. However, aberrant Wnt signaling has also been observed as a significant pathway in many cancers^44^. From Fig. 6, we have also observed that the top 75 discriminating genes are associated with numerous adenocarcinomas and squamous cell carcinomas.

## Conclusion

Classification of cancer types using machine learning algorithms leverages diagnostic capabilities of conventional clinical and morphological approaches. This article employed regularized multi-task learning (RMTL) to perform pan-cancer classification using RNASeq gene expressions data. We have prepared the pan-cancer classification dataset by accumulating gene expressions of highly significant differentially expressed (DE) genes for each cancer.

We have compared the performance of our proposed PC-RMTL model with SVM-Lin, SVM-RBF, kNN, RF, and DT through precision, recall, f1-score, MCC, ROC curve, precision–recall curve, and logistic loss. Even though the pan-caner dataset is highly imbalanced due to considerable differences in the number of samples across the cancer types, the PC-RMTL outperforms all the other five classifiers (PC-RMTL achieves 96.07% accuracy and 95.80% MCC score). We have observed that PC-RMTL performs exceptionally well in distinguishing all of the pan-cancer samples except the READ. To the best of our knowledge, this is a significant improvement over the existing works in pan-cancer classification as they have failed to classify many cancer types from one another reliably.

Furthermore, we have identified the key discriminating features (genes) in pan-cancer classification using the coefficients (weights) of the trained SVM-Lin classifier and an independent features (genes) selection algorithm: MRMR. Pan-cancer classification with all the classification models using those small sets of discriminating features has also been carried out. Interestingly, the PC-RMTL again outperformed all the classifiers with these small feature sets. The top discriminating genes are evaluated using functional enrichment analysis. It has been discovered that almost all of the top discriminating genes are highly associated with cancer-related gene ontology (GO) terms and pathways.

Since our classification model has reliably classified all cancer types, it can be utilized to design diagnostic kits for classifying cancer samples from liquid biopsy, prognosis, and forecasting of cancers. A thorough study of the cancer-specific features and their correlation across several cancer types will certainly unveil path-breaking insights into the understanding, prognosis, efficient therapeutics for cancers.

## Supplementary information

Supplementary Tables.
