# Supplementary material for: Pan-cancer classification by regularized multi-task learning
Source: Sci Rep. 2021 Dec 20;11:24252. doi: 10.1038/s41598-021-03554-8 (PMC8688544; doi:10.1038/s41598-021-03554-8)
Supplement: Supplementary file 1 — Supplementary Tables. [file 41598_2021_3554_MOESM1_ESM.pdf]

Title: **Pan-cancer classification by regularized multi-task learning**

Authors:

Sk Md Mosaddek Hossain[1, *], Lutfunnesa Khatun [2], Sumanta Ray[1], and Anirban Mukhopadhyay [2]

[1] Department of Computer Science and Engineering, Aliah University, Kolkata, India, 700160.

[2] Department of Computer Science and Engineering, University of Kalyani, Kalyani, India, 741235.

*mosaddek.hossain@gmail.com

Table S1: Table shows the precision, recall, f1-score of the SVM-Lin, SVM-RBF, RF, KNN and DT classifiers.

	SVM-Lin			SVM-RBF			RF			KNN			DT		
**Cancer**	**Precision**	**Recall**	**F1-score**	**Precision**	**Recall**	**F1-score**	**Precision**	**Recall**	**F1-score**	**Precision**	**Recall**	**F1-score**	**Precision**	**Recall**	**F1-score**
BLCA	0.9750	0.9398	0.9571	0.9630	0.9398	0.9512	0.8916	0.8916	0.8916	0.9146	0.9036	0.9091	0.7174	0.7952	0.7543
BRCA	0.9908	0.9818	0.9863	0.9954	0.9818	0.9886	0.9817	0.9773	0.9795	1.0000	0.9727	0.9862	0.9486	0.9227	0.9355
CESC	0.9672	0.9672	0.9672	0.9833	0.9672	0.9752	0.9107	0.8361	0.8718	0.9180	0.9180	0.9180	0.6286	0.7213	0.6718
CHOL	1.0000	0.8571	0.9231	0.8571	0.8571	0.8571	0.8000	0.5714	0.6667	0.7500	0.8571	0.8000	0.2500	0.1429	0.1818
COAD	0.9010	0.9479	0.9239	0.8846	0.9583	0.9200	0.7328	1.0000	0.8458	0.7460	0.9792	0.8469	0.6514	0.7396	0.6927
ESCA	0.9000	0.8438	0.8710	0.9000	0.8438	0.8710	0.9546	0.6563	0.7778	0.8077	0.6563	0.7241	0.6154	0.7500	0.6761
GBM	1.0000	1.0000	1.0000	1.0000	1.0000	1.0000	1.0000	1.0000	1.0000	0.9688	1.0000	0.9841	0.9688	1.0000	0.9841
HNSC	0.9612	0.9900	0.9754	0.9612	0.9900	0.9754	0.9074	0.9800	0.9423	0.8952	0.9400	0.9171	0.7615	0.8300	0.7943
KICH	0.8125	1.0000	0.8966	0.8125	1.0000	0.8966	0.7222	1.0000	0.8387	0.7222	1.0000	0.8387	1.0000	0.6154	0.7619
KIRC	0.9619	0.9352	0.9484	0.9712	0.9352	0.9528	0.9709	0.9259	0.9479	0.9623	0.9444	0.9533	0.8909	0.9074	0.8991
KIRP	0.9273	0.8793	0.9027	0.9286	0.8966	0.9123	0.9286	0.8966	0.9123	0.9623	0.8793	0.9189	0.8846	0.7931	0.8364
LIHC	1.0000	0.9189	0.9578	1.0000	0.8919	0.9429	1.0000	0.9189	0.9578	1.0000	0.8784	0.9353	0.9672	0.7973	0.8741
LUAD	0.9626	0.9626	0.9626	0.9623	0.9533	0.9578	0.9196	0.9626	0.9406	0.9346	0.9346	0.9346	0.9278	0.8411	0.8824
LUSC	0.9694	0.9500	0.9596	0.9500	0.9500	0.9500	0.9674	0.8900	0.9271	0.8900	0.8900	0.8900	0.7813	0.7500	0.7653
PAAD	0.8974	1.0000	0.9460	0.8750	1.0000	0.9333	0.9167	0.9429	0.9296	0.8750	1.0000	0.9333	0.9091	0.8571	0.8824
PCPG	0.9730	1.0000	0.9863	1.0000	1.0000	1.0000	0.9474	1.0000	0.9730	0.9730	1.0000	0.9863	0.9211	0.9722	0.9460
PRAD	0.9900	0.9900	0.9900	0.9901	1.0000	0.9950	0.9709	1.0000	0.9852	0.9709	1.0000	0.9852	0.9615	1.0000	0.9804
READ	0.8214	0.6970	0.7541	0.8462	0.6667	0.7458	0.0000	0.0000	0.0000	0.7143	0.1515	0.2500	0.3125	0.3030	0.3077
SARC	0.8525	1.0000	0.9204	0.8667	1.0000	0.9286	0.8387	1.0000	0.9123	0.8621	0.9615	0.9091	0.7667	0.8846	0.8214
SKCM	1.0000	1.0000	1.0000	1.0000	1.0000	1.0000	1.0000	1.0000	1.0000	1.0000	1.0000	1.0000	1.0000	0.8500	0.9189
STAD	0.9231	0.9600	0.9412	0.9231	0.9600	0.9412	0.8588	0.9733	0.9125	0.9296	0.8800	0.9041	0.8406	0.7733	0.8056
STN	0.9603	0.9528	0.9565	0.9524	0.9449	0.9486	0.9500	0.8976	0.9231	0.9147	0.9291	0.9219	0.8197	0.7874	0.8032
Accuracy			0.9541			0.9528			0.9203			0.9190			0.8259
Macro avg	0.9430	0.9442	0.9421	0.9374	0.9426	0.9383	0.8714	0.8782	0.8698	0.8960	0.8944	0.8839	0.7966	0.7743	0.7807
Weighted avg	0.9551	0.9541	0.9539	0.9540	0.9528	0.9525	0.9075	0.9203	0.9109	0.9205	0.9190	0.9140	0.8321	0.8259	0.8269

Table S2: Table shows the accuracy of the different classifiers using different no of genes as selected features through the MRMR [1] algorithm.

No Genes	PC-RMTL	SVM-Lin	SVM-RBF	RF	KNN	DT
100	0.9207	0.9126	0.9152	0.9037	0.8878	0.8157
200	0.9360	0.9298	0.9311	0.9107	0.9043	0.8246
300	0.9410	0.9369	0.9388	0.9171	0.9177	0.8227
400	0.9455	0.9426	0.9458	0.9158	0.9165	0.8240
500	0.9512	0.9445	0.9458	0.9165	0.9120	0.8195
600	0.9543	0.9413	0.9483	0.9228	0.9094	0.8259
700	0.9575	0.9464	0.9496	0.9190	0.9101	0.8291
800	0.9588	0.9490	0.9509	0.9260	0.9139	0.8342
900	0.9581	0.9528	0.9515	0.9247	0.9133	0.8316
1000	0.9613	0.9541	0.9534	0.9216	0.9203	0.8444

[1] Peng, H., Long, F. & Ding, C. Feature selection based on mutual information criteria of max-dependency, max-relevance,and min-redundancy.IEEE Transactions on Pattern Analysis Mach. Intell.27, 1226–1238, DOI: 10.1109/TPAMI.2005.159(2005).

Table S3: Table shows gene ontology terms (biological processes), KEGG pathways, and disease-genes associations of the top 75 discriminating genes.

GO Term	p-value	KEGG Pathway	p-value	Disease-Genes Association	p-value
regulation of cellular senescence (GO:2000772)	9.69E-05	Melanogenesis	0.00648805	Adenocarcinoma	3.64E-08
extracellular matrix organization (GO:0030198)	2.20E-04	Pathways in cancer	0.014520467	Fibroadenoma	1.30E-07
positive regulation of cell aging (GO:0090343)	3.83E-04	Wnt signaling pathway	0.021585719	Neoplasm Metastasis	3.71E-07
heterochromatin assembly (GO:0031507)	3.83E-04	Hepatocellular carcinoma	0.025302336	Secondary malignant neoplasm of liver	3.87E-07
replicative senescence (GO:0090399)	7.47E-04	Glioma	0.03228684	Secondary malignant neoplasm of lymph node	7.55E-07
epithelium development (GO:0060429)	9.21E-04	Calcium signaling pathway	0.033703919	Squamous cell carcinoma	8.37E-07
DNA strand elongation (GO:0022616)	0.001053695	Salivary secretion	0.044961712	Stomach Carcinoma	3.35E-06
regulation of stem cell proliferation (GO:0072091)	0.001609305	GnRH signaling pathway	0.047685326	Mammary Neoplasms	3.58E-06
mitotic G2 DNA damage checkpoint (GO:0007095)	0.001819459	Human T-cell leukemia virus 1 infection	0.049232625	Anaplastic thyroid carcinoma	4.35E-06
ureteric bud morphogenesis (GO:0060675)	0.002523463	Human cytomegalovirus infection	0.052580269	Tumor Progression	5.05E-06
